# Monomeric a-synuclein (aS) inhibits amyloidogenesis of human prion protein (hPrP) by forming a stable aS-hPrP hetero-dimer.

**DOI:** 10.1080/19336896.2021.1910176

**Published:** 2021-04-14

**Authors:** Satoshi Yamashita, Yuji O. Kamatari, Ryo Honda, Ayumi Niwa, Hiroyuki Tomiata, Akira Hara, Kazuo Kuwata

**Affiliations:** a United Graduate School of Drug Discovery and Medical Information Sciences, Gifu University, Tokai National Higher Education and Research System, Gifu, Japan; b Institute for Glyco-core Research, Tokai National Higher Education and Research System, Gifu, Japan; c Department of Tumor Pathology, Gifu University Graduate School of Medicine, Tokai National Higher Education System, Gifu, Japan; d Department of Gene and Development, Gifu University School of Medicine, Tokai National Higher Education and Research System, Gifu, Japan

**Keywords:** α-synuclein, prion, hetero-oligomer, pathogenic conversion, AFM

## Abstract

Intermolecular interaction between hPrP and αS was investigated using high-speed atomic force microscopy, dynamic light scattering, and nuclear magnetic resonance. We found that hPrP spontaneously gathered and naturally formed oligomers. Upon addition of monomer αS with a disordered conformation, poly-dispersive property of hPrP was lost, and hetero-dimer formation started quite coherently, and further oligomerization was not observed. Solution structure of hPrP-αS dimer was firstly characterized using hetero-nuclear NMR spectroscopy. In this hetero-dimeric complex, C-terminal helical region of hPrP was in the molten-globule like state, while specific sites including hot spot and C-terminal region of αS selectively interacted with hPrP. Thus αS may suppress amyloidogenesis of hPrP by trapping the hPrP intermediate by the formation of a stable hetero-dimer with hPrP.

**Abbreviations:** hPrP, human prion protein of amino acid residues of 23-231; PrP^C^, cellular form of prion protein; PrP^Sc^, scrapie form of prion protein, HS-AFM; high speed atomic force microscopy; αS, α-synuclein; DLS, dynamic light scattering

## Introduction

Intermolecular interaction is a key pathological event in protein misfolding disease [1,2]. Amyloid deposits are mainly composed of a specific disease-associated amyloidogenic protein, such as amyloid-β (Aβ) in Alzheimer’s disease [3], α-synuclein (αS) in Parkinson’s disease [4], and prion protein (PrP) in transmissible spongiform encephalopathy [5]. Recent evidence has indicated that several amyloidogenic proteins can interact with a different disease-associated protein to dramatically impact on the development of amyloid deposits [6]. Cross-seeding is also a prominent example of the cross-interactions, by which an amyloid fibril composed of one specific protein can promote amyloidogenesis of a different amyloidogenic protein [7].

On the other hand, specific interactions between amyloidogenic proteins were reported. Lauren et al [8]. suggested that cellular prion protein (PrP^C^) on cell surface serves as a receptor for oligomeric amyloid β(Aβ) and blocks the long term potentiation (LTP)　upon Aβ binding, although it was reported later that Aβ42 blocks LTP even without PrP^C^ [9]. Selective inhibition of amyloidogenic proteins against amyloidogenesis was also reported, such as chaperone-like activity of α-synuclein (αS) [10–14], which may lead to the development of therapeutics.

We previously confirmed that monomeric αS suppressed amyloid formation of human prion protein (hPrP) [14]. Although α-synuclein (αS) amyloids reportedly interacts with hPrP and blocks prion replication [15], in our experiment Aβ amyloid was strongly bound with hPrP but αS amyloid [15]. However, structural evidence of the direct interaction between monomeric αS and hPrP was still elusive.

The present study aimed to characterize the intermolecular interaction between hPrP and monomeric αS in molecular shape resolution as well as in atomic resolution. To do this, we initially applied high-speed atomic force microscopy (HS-AFM) [16] for the observation of the real-time molecular shape alteration of hPrP and αS upon mixing. HS-AFM was useful for the observation of protein aggregation process [17], particularly for the real-time observation as presented here. We then measured dynamic light scattering (DLS) [18,19] for resolving the population of conformers in a mixture of hPrP and αS. Finally, we measured various ^1^H-^15^N HSQC spectra to elucidate the conformational characteristics of the hPrP-αS complex, and discussed the possible inhibitory mechanism of αS upon hPrP pathogenic conversion reaction.

## Results

### Dispersive oligomer configurations of hPrP

**Figure 1.**
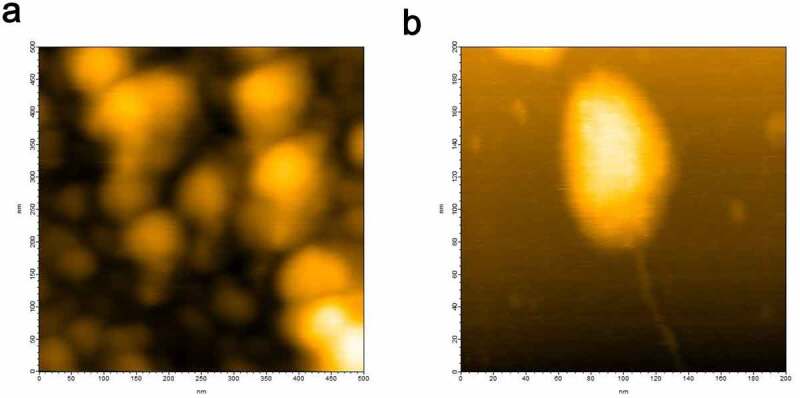
(a) HS-AFM image of hPrP showing various heterogeneous oligomeric states distributed from monomers to hexamers. (b) Typical hexameric oligomer of hPrP with a tail

HS-AFM imaging of hPrP at a concentration of 30 nM revealed that particle diameters of hPrP were distributed from 2 nm to 100 nm corresponding to monomer to various sizes of oligomers, and generally consisted of a head and tail (Figure 1a). Height analysis of a large oligomer (Figure 1b) revealed that the diameter of its body was around 8 nm and its tail was 2 nm. According to AFM calibration [20], these corresponded to hexamers and monomers, respectively. Head & tail conformer with a variety of sizes (Figs. S1 and S2) suggests that oligomer growth may not follow an amyloid growth model [21,22] in which monomers continue attaching to seeds to form larger particles. Instead, these basic growth unit collide and coalesce to create larger oligomers [20]. These inter-oligomer interactions may be mediated by thin fibrils attached to oligomer (Fig. S2).

## Disordered conformation of αS monomer

**Figure 2.**
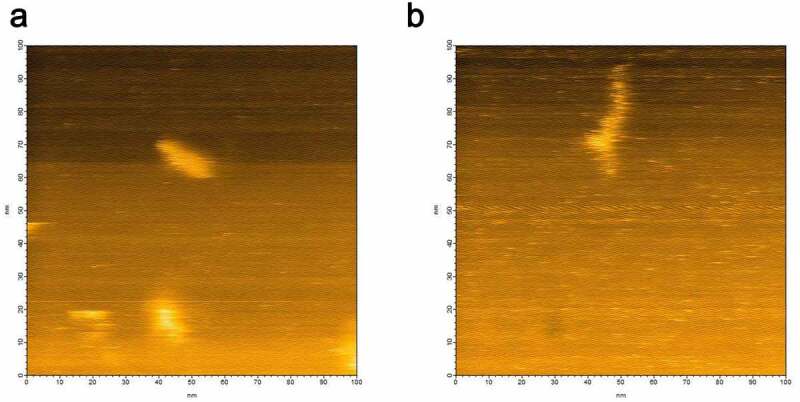
HS-AFM images of αS showing (a) globular conformation and (b) extended conformation of αS

**Figure 3.**
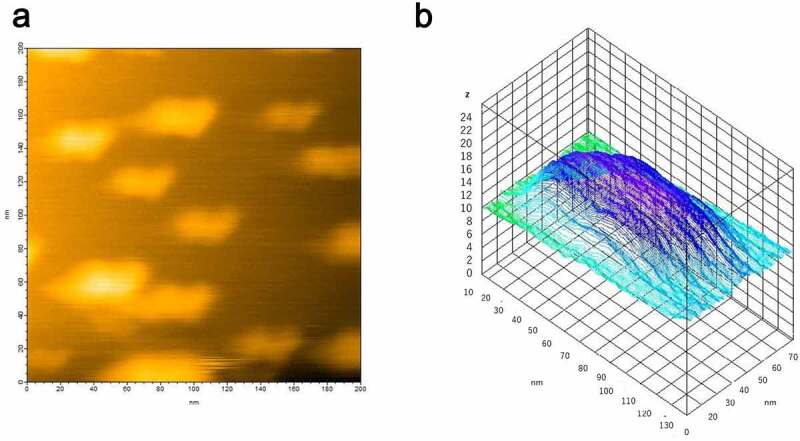
(a) HS-AFM image of hPrP and αS complex at equilibrium. (b) Height analysis shows the early complex is hetero-dimer of hPrP and αS. HS-AFM image of hPrP and αS complex at equilibrium. Height analysis showed the oligomer at equilibrium was a trimer

HS-AFM images of αS are shown in Figure 2. The conformation of αS changed from almost globular (Figure 2a) to partially extended (Figure 3b), and we observed further variations in conformation [23] (data not shown). Thus, αS predominantly existed as a disordered state [24,25].

## Interaction between hPrP and αS

The heterogeneous hPrP oligomeric configurations were confirmed after loading 1 µl of hPrP (30 nM) onto the mica surface attached on the glass pole of the HS- AFM apparatus. Then 1 µl of αS (30 nM) was gently poured onto the mica surface. Heterogeneous oligomers of hPrP were quickly dissolved and particles with a transient shape appeared coherently within 1 frame of the HS-AFM apparatus (5 sec. in this experiment) (Fig. S3), and finally uniformly distributed dimers were shown. The height of this complex was about 2 nm (Figure 3b). In equilibrium, monomeric structure corresponding to hPrP or αS, or oligomer larger than dimer was not observed. Thus this complex was considered to be hetero-dimer of hPrP and αS. No further change was observed after hetero-dimer formation.

## Population shifts detected by dynamic light scattering (DLS)

**Figure 4.**
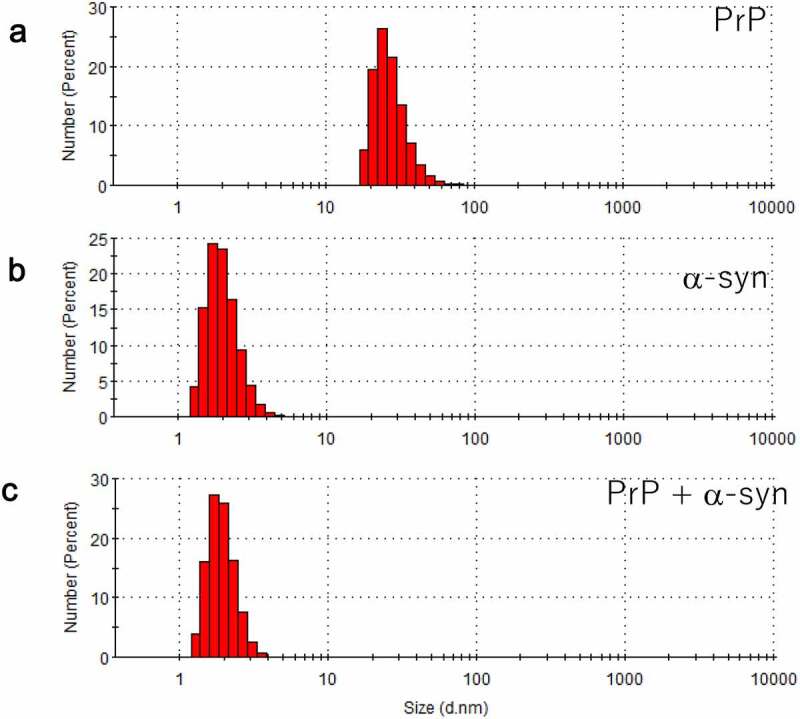
Dynamic light scattering (DLS) measurement of a number (population) of particles as a function of particle size (diameter) in (a) hPrP solution with peak at 24 nm, corresponding to an oligomer, (b) αS solution with a diameter of 1.7 nm, corresponding to a monomer, and (c) a mixture of hPrP and αS with a peak at the diameter of 1.7 nm corresponding to a hetero-dimer of hPrP and αS

While AFM is sensitive to molecular shape, it is not good at evaluating population. Therefore, DLS was used hereas a complementary measurement. We initially observed that the hPrP solution was mainly populated with particles with a diameter of 24 nm (Figure 4a), suggesting that hPrP predominantly existed as oligomers. Since hPrP oligomers consist of a head and tail (Figure 1), the diffusion constant would become much slower than that of hexamer with spherical shape.

Particles with a diameter of 1.7 nm were predominantly populated in the αS solution (Figure 4b). Because theoretically calculated hydrodynamic radius of αS assuming spherical particle is 0.7 nm, the observed diameter indicated that αS predominantly existed as a monomer. Since αS is natively unfolded (Figure 2), it is reasonable that the observed diffusion constant was slower than that of spherical particle.

When hPrP and αS were mixed in a one-to-one molecular ratio in solution, the oligomer peak of hPrP disappeared, and a peak at a diameter of 1.7 nm was detected (Figure 4c), which corresponds to the stable hetero-dimer of hPrP and αS (Figure 3). It should be noted that this hetero-dimer were compact with no apparent tail (disordered region) (Figure 3). Thus, the diffusion constant of monomeric αS and that of the compact hetero-dimer showed a close hydrodynamic radius.

## Conformational characteristics of heterodimer of hPrP and αS

**Figure 5.**
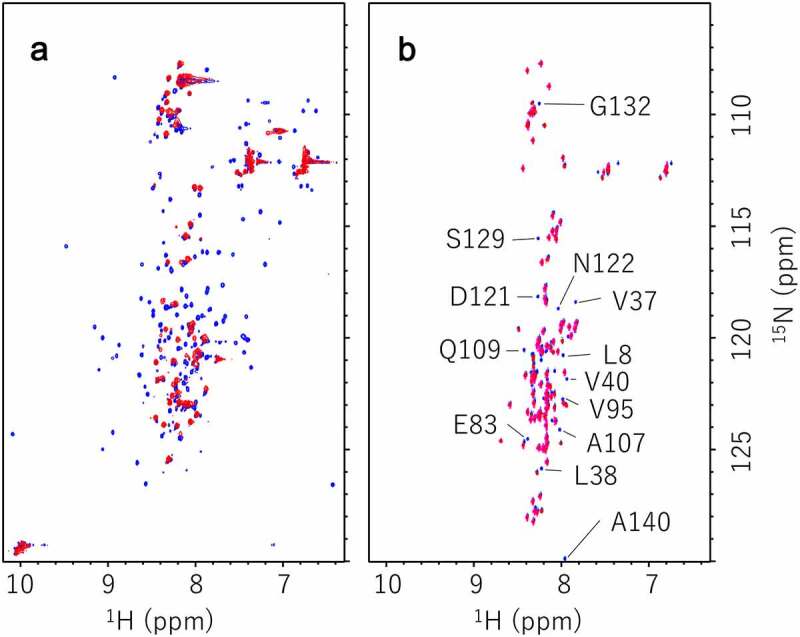
(a) [1H-15]N HSQC spectra of 200 µM [15]N labelled hPrP without (blue) or with 220 µM non-labelled αS (red) at pH 6.1 in 99% H_2_O/1% D_2_O. (b) [1H-15]N HSQC spectra of 206 µM [15]N labelled αS without (blue) or with 173 µM non-labelled hPrP (red) at pH 4.6 in 99% H_2_O/1% D_2_O

We prepared four kinds of proteins for NMR, i.e. hPrP with or without [15]N label, and αS with or without [15]N label. Figure 5a shows the ^1^H-^15^N HSQC spectra of ^15^N labelled hPrP without (blue) or with non-labelled αS (red). Apparently, signals from the C-terminal half region of native hPrP were mostly disappeared and the remained peaks were broadened upon the interaction with αS, suggesting the conformational conversion from native to the molten globule state. Figure 5b shows the ^1^H-^15^N HSQC spectra of ^15^N labelled αS without (blue) or with non-labelled hPrP (red). Although native αS was disordered state (IDP) [26], chemical shift of thirteen residues, i.e. L8, V37, L38, V40, E83, V95, A107, Q109, D121, N122, S129, G132, and A140 were selectively shifted upon binding of hPrP, suggesting the specific contact regions in αS. We plotted chemical shift alterations and the peak volume changes in αS upon binding with hPrP in Fig. S4, indicating that chemical shifts in αS around residues ~ 40 and ~ 60 were strongly perturbed (Figs. S4A & S4B), and peak intensities of C-terminal regions (residues 105 ~ 120) were markedly reduced (Fig. S4C).

## Discussion

**Figure 6.**
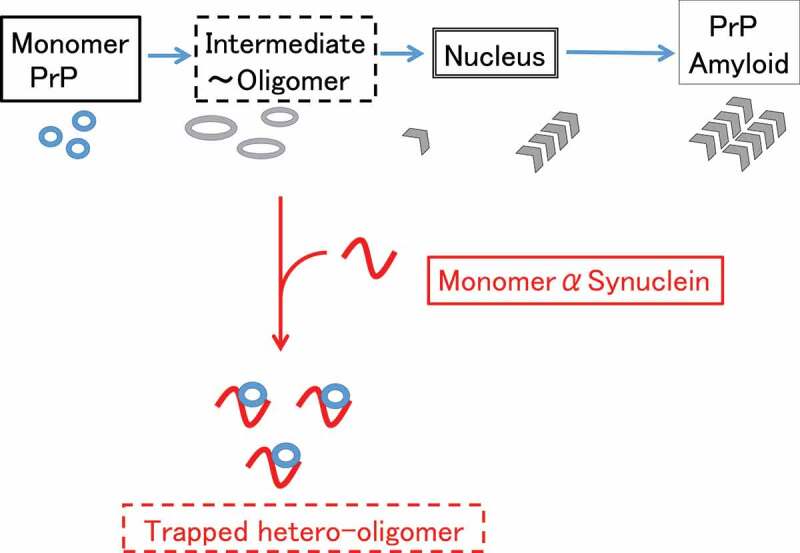
Possible mechanism of suppression of the pathogenic conversion of hPrP by αS. Monomeric αS can trap the intermediate state hPrP and forms a stable hetero-dimer, depleting the available monomeric hPrP for pathogenic conversion

We previously reported that monomeric αS can suppress amyloidogenesis of PrP [14], but neither bind to fully folded PrP nor fibrillar PrP [14]. Therefore we hypothesized that monomeric αS binds to the hydrophobic region of partially unfolded PrP to prevent further structural changes toward misfolded aggregates [14] . This hypothesis suggests the monomer αS can trap a partially unfolded (or molten globule) state of PrP which tends to oligomerize (Figure 6).

In the present study, we found that monomer αS dissolved heterogenous hPrP oligomer into a compact dimer complex (Figure 3). This hetero-dimer of hPrP and αS was rather uniform and stable, and the stable dimer formation would efficiently deplete the available monomeric hPrP molecule for pathogenic conversion [27], resulting in the suppression of misfolding of PrP and further pathogenic conformational conversion. This finding can be linked to prior research showing the chaperone-like activity of monomeric αS to suppress the misfolding of a number of different proteins both *in* *vivo* and *in* *vitro* [11,28–30].

Conformation of stable hetero-dimer of hPrP and αS was characterized using NMR spectra. hPrP underwent the transition from native conformation to the molten-globular state upon binding with αS. In contrast in αS, chemical shifts of the signals from regions including the hot spot (amino acid residue number of 38–45), amino acid residue number of ~ 60 and the C-terminal region (amino acid residue number of 120–140) were selectively altered upon the interaction with hPrP, suggesting the specific interaction. Intriguingly, these regions are largely included in those involved in the intermolecular interaction with αS or βS elucidated using PRE experiments [31].

Although further studies using a kinetic methods may be required to elucidate the detailed interaction between αS and partially folded proteins [32], our results suggest a possible involvement of αS in transmissible spongiform encephalopathy pathology. It was recently shown that knockout of the αS gene did not affect the incubation period of prion propagation in mouse brain [33]. Since αS chaperone interacts with partially folded proteins or oligomer, and suppresses the nucleation step of amyloid formation, αS may suppress the spontaneous generation, or evolution [34], but not propagation of infectious prion. Although there reported the interaction between αS and PrP using the immunoprecipitation and merging by immunofluorescence *in vivo* [15], there has been no corresponding *in vivo* results showing the selective inhibition of nucleation step thus far.

## Materials and methods

A plasmid encoding human PrP, amino acid residues 23–230 [hPrP] was prepared according to a previously reported protocol [35]. A recombinant hPrP was expressed using an *E. coli* expression system and purified to homogeneity according to the previously published protocol [35].

A pT7-7 vector encoding wild-type human αS (residues 1–140) was purchased from Addgene (code 36,046) [14]. Recombinant human αS was expressed using ECOS™ competent *E. coli* BL21 (DE3) (Nippongene, code 312–06534) and purified to homogeneity according to the previously published protocol [14].

Tapping mode high-speed atomic force microscopy (HS-AFM, NanoExplorer, Research Institute of Biomolecule Metrology Co., Ltd, Tsukuba, Japan) was performed at room temperature in aqueous solution using a small cantilever (BL-AC10FS-A2, Olympus) with a spring constant *k* ∼0.1 N/m and resonance frequency *f* = 1.5 MHz. hPrP and αS solutions (20 mM phosphate [pH 7.0]) were introduced into a sample chamber for HS-AFM. Image sequences were processed using ImageJ software (imagej.nih.gov/ij/).

To observe the interaction between hPrP and αS, 2 µL of hPrP (0.1 µM) solution was mounted onto the mica surface on the glass pole and stayed for 10 min. and mounted again. We confirmed the clear images of the dispersive hPrP conformations, and then added 2 µL of αS (0.1 µM), and a movie was recorded with the time resolution of 1–5 s.

DLS was performed using a Zetasizer NanoS (Malvern Panalytical, Egham, Surrey, UK). 100 µl of hPrP solution (100 µM) and 100 µl of αS solution (100 µM) were used for characterizing the population in each solution. For observing the interaction between hPrP and αS, we mixed 50 µl of hPrP solution (100 µM) and the same volume of αS solution (100 µM) and measured immediately.

NMR experiments were carried out using Avance 800 spectrometer (Bruker BioSpin) operating at a ^1^H frequency of 800.15 MHz and a ^15^N frequency of 81.08 MHz. A 5 mm ^1^H inverse detection cryogenic probe with triple-axis gradient coils was used for all measurements. ^1^H–^15^N HSQC spectra were acquired at 25°C, with 4,096 complex points covering 12,821 Hz for ^1^H and 256 complex points covering 2,108 Hz for ^15^N. NMR data were processed using the TopSpin NMR software package (Bruker BioSpin).

## Supplementary Material

Supplemental Material
