# Supplementary material for: Monomeric a-synuclein (aS) inhibits amyloidogenesis of human prion protein (hPrP) by forming a stable aS-hPrP hetero-dimer
Source: Prion. 2021 Apr 14;15(1):37–43. doi: 10.1080/19336896.2021.1910176 (PMC8049198; doi:10.1080/19336896.2021.1910176)
Supplement: Supplemental Material [file KPRN_A_1910176_SM5117.pptx]

## Slide 1
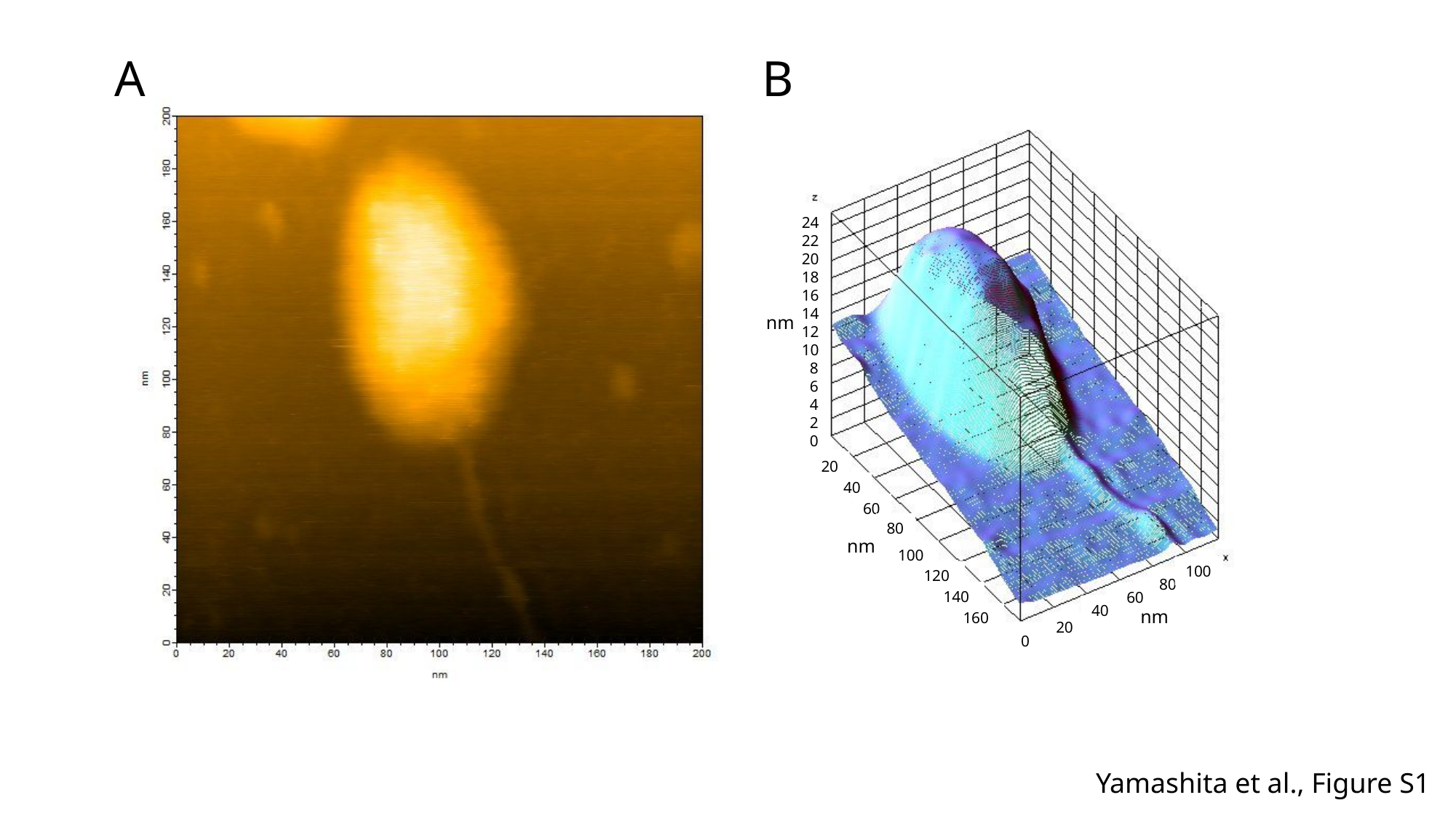

A
B
24
22
20
18
16
14
12
10
 8
 6
 4
 2
 0
nm
20
40
60
80
nm
100
100
120
80
140
60
40
nm
160
20
0
Yamashita et al., Figure S1

## Slide 2
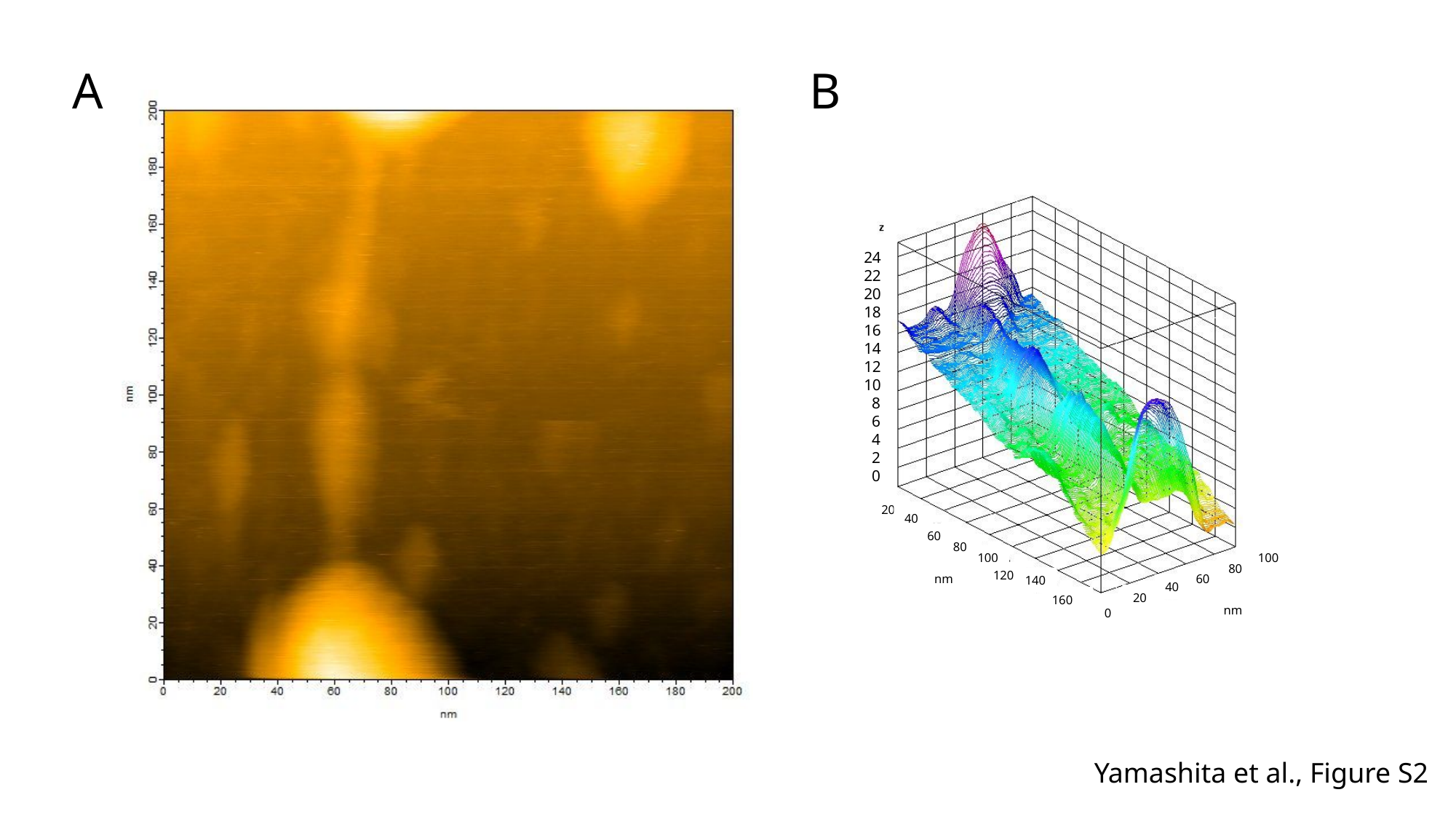

A
B
24
22
20
18
16
14
12
10
 8
 6
 4
 2
 0
20
40
60
80
100
100
80
120
nm
60
140
40
20
160
nm
0
Yamashita et al., Figure S2

## Slide 3
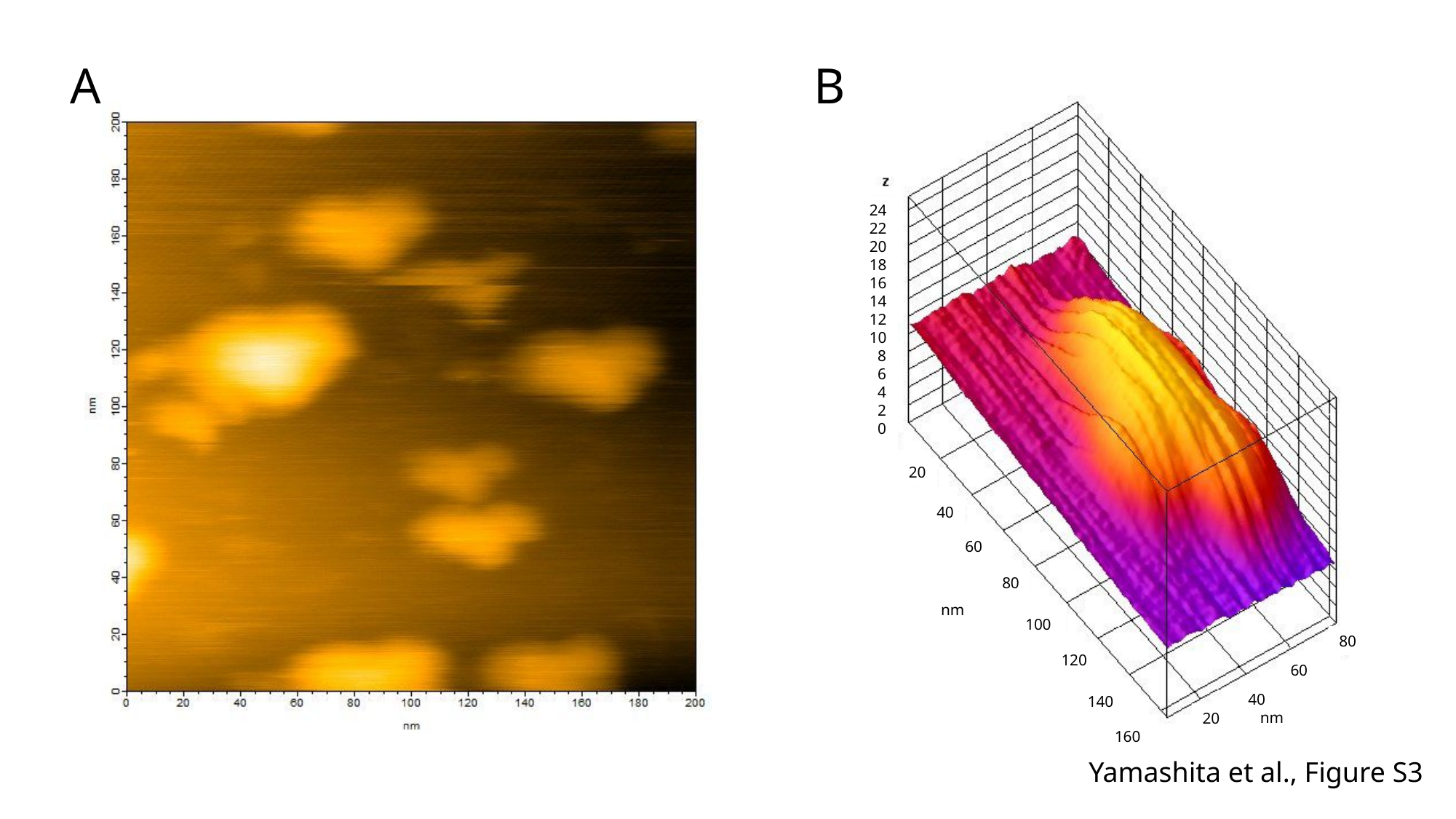

B
A
24
22
20
18
16
14
12
10
 8
 6
 4
 2
 0
20
40
60
80
nm
100
80
120
60
40
140
nm
20
160
Yamashita et al., Figure S3

## Slide 4
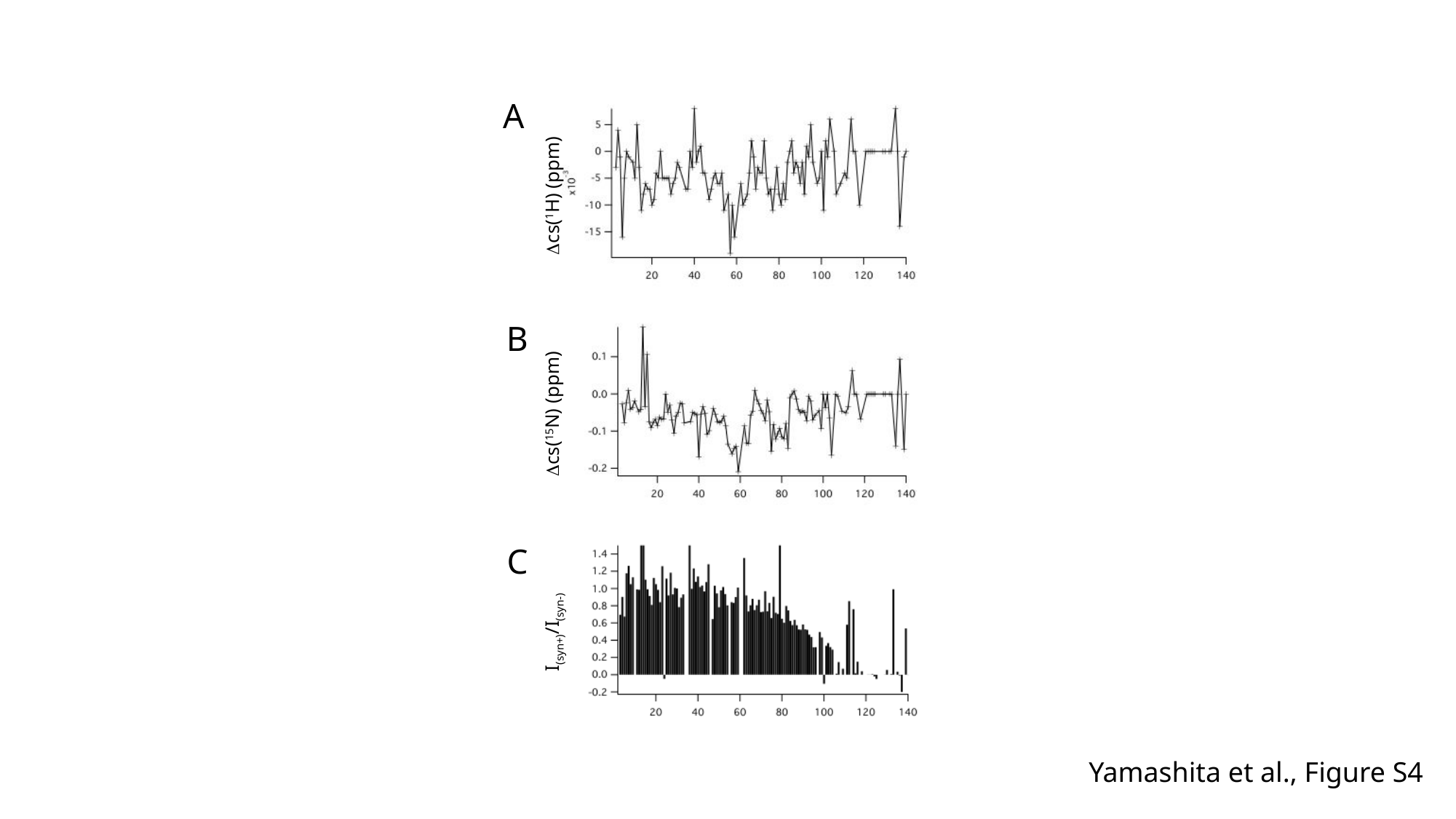

A
Dcs(1H) (ppm)
B
Dcs(15N) (ppm)
C
I(syn+)/I(syn-)
Yamashita et al., Figure S4
